# Home Use of a Neural-connected Sensory Prosthesis Provides the Functional and Psychosocial Experience of Having a Hand Again

**DOI:** 10.1038/s41598-018-26952-x

**Published:** 2018-06-29

**Authors:** Emily L. Graczyk, Linda Resnik, Matthew A. Schiefer, Melissa S. Schmitt, Dustin J. Tyler

**Affiliations:** 1 Department of Biomedical Engineering, Case Western Reserve University, Cleveland, OH USA; 2 Providence Veterans Affairs Medical Center, Providence, RI USA; 3 Health Services, Policy and Practice, Brown University, Providence, RI USA; 4 Louis Stokes Cleveland Veterans Affairs Medical Center, Cleveland, OH USA

## Abstract

The loss of a hand has many psychosocial repercussions. While advanced multi-articulated prostheses can improve function, without sensation, they cannot restore the full experience and connection of a hand. Direct nerve interfaces can restore naturalistic sensation to amputees. Our sensory restoration system produced tactile and proprioceptive sensations on the hand via neural stimulation through chronically implanted electrodes. In this study, upper limb amputees used a sensory-enabled prosthesis in their homes and communities, autonomously and unconstrained to specific tasks. These real-life conditions enabled us to study the impact of sensation on prosthetic usage, functional performance, and psychosocial experience. We found that sensory feedback fundamentally altered the way participants used their prosthesis, transforming it from a sporadically-used tool into a readily and frequently-used hand. Functional performance with sensation improved following extended daily use. Restored sensation improved a wide range of psychosocial factors, including self-efficacy, prosthetic embodiment, self-image, social interaction, and quality of life. This study demonstrates that daily use of a sensory-enabled prosthesis restores the holistic experience of having a hand and more fully reconnects amputees with the world.

## Introduction

A hand serves many roles, acting as a multifunctional tool for interacting with the world, serving as a means of connecting emotionally and socially with others, and expressing or representing feelings, thoughts, or symbolic aspects of one’s self^1^. Consequently, when a person loses a hand due to amputation, he or she may experience numerous physical and psychosocial challenges, including losses in body image, self-confidence and self-concept, pain, disruptions in employment and lifestyle, impairments in functional capabilities, and stresses on relationships and social integration^2–5^. In fact, 25–35% of persons living with limb loss suffer from significant depressive symptoms, a prevalence that is more than three times that of the general population^2,6–9^.

Prosthetic rehabilitation historically has focused on restoring physical function, with little attention, until recently, on the psychosocial aspects of rehabilitation^10,11^. Prosthetics research and industry advances have focused on improving the functionality of hand prostheses, introducing increasingly complex anthropomorphic designs with multiple degrees of freedom and advanced control strategies^12–15^. Yet these efforts may not address the needs of those living with limb loss, since a recent meta-analysis found that psychosocial outcomes are more important to disabled individuals and more strongly related to quality of life than functional impairment^16^.

Restoring the sensation of the missing hand can mitigate the psychosocial deficits associated with limb loss^17,18^. Tactile and proprioceptive feedback are highly desired by the myoelectric prosthetics community, since without them, prosthesis users must rely on visual observation, listening to the hand motors, or feeling sensations on their residual limb through the socket^19,20^. However, there are currently no commercially available prostheses that provide intuitive sensory feedback to wearers, wherein sensors on the prosthesis trigger tactile and proprioceptive sensations that are perceived to be collocated with the missing hand.

To bridge this gap, several research groups have investigated direct nerve interfaces to provide sensory feedback to the prosthesis. Nerve stimulation through implanted electrodes provides sensations in small, functionally-relevant locations on the missing hand and fingers, that are appropriate in perceived magnitude, and that are tactile and proprioceptive in modality^21–23^. We, and others, have shown that this restored sensation can aid in object identification and manipulation tasks when using a sensorized prosthesis^18,24–26^. However, all demonstrations of success in these stereotyped tasks have been in a constrained, researcher-supervised laboratory environment. It is not known if these results will translate to the realistic conditions of everyday life or whether myoelectric prosthesis users will indeed find the provided feedback useful and desirable. Furthermore, the impact of sensory feedback on psychosocial outcomes, such as in interpersonal interactions or perceived disability, remains mostly unexplored. The important long-term psychosocial benefits of sensation cannot be measured when usage is limited to relatively short laboratory sessions and without the participants having choice in when and how they use the prosthesis. Participants need to be able to use a sensory restoration prosthesis as they deem relevant to their individual lives and in real-life settings for researchers to better understand the impact of sensory feedback on persons with limb loss.

**Figure 1:**
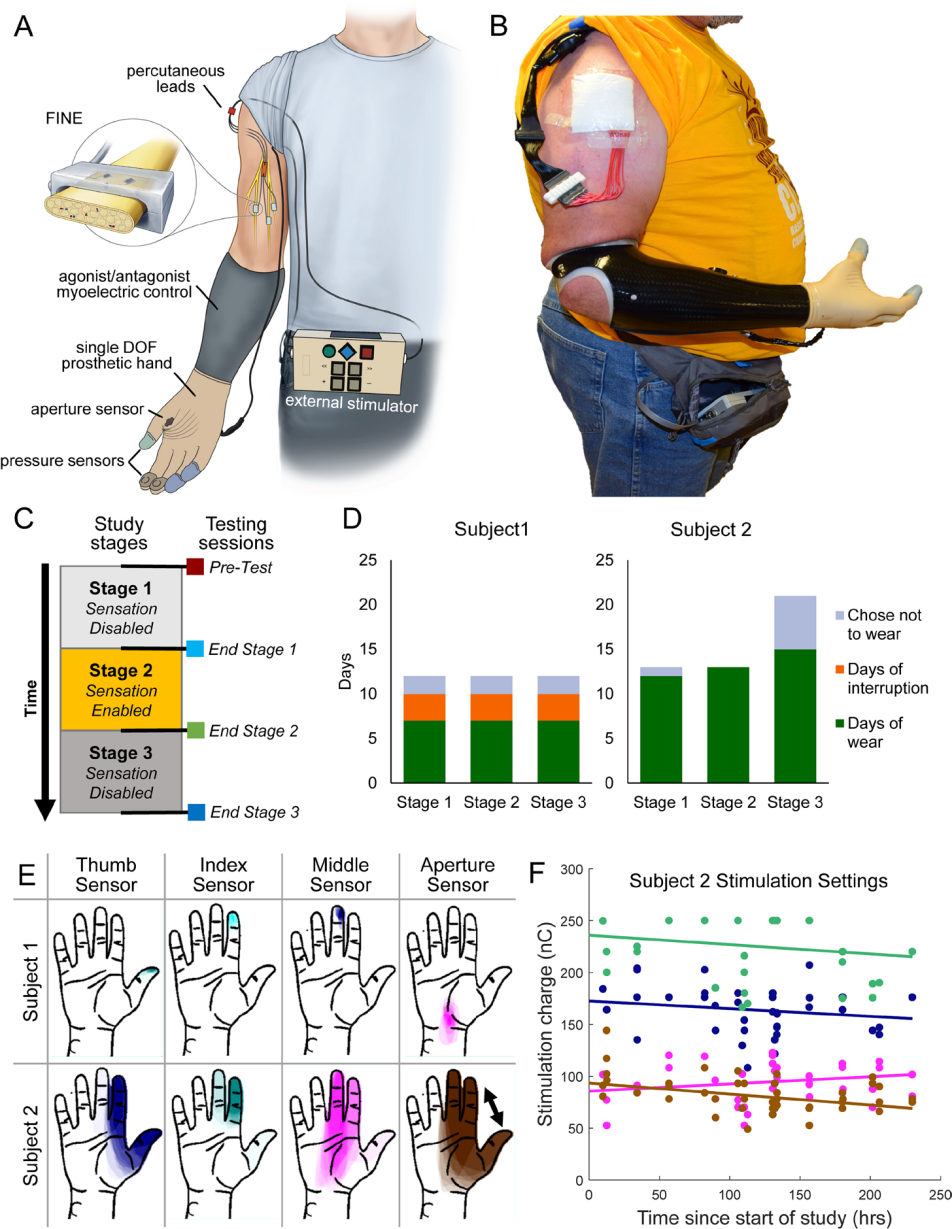
Take home sensory restoration system. (**A**) Participants wore their own prosthetic socket with standard agonist/antagonist myoelectric control. They were provided a VariPlus Speed™ prosthetic hand (Ottobock, Vienna, Austria), which had been augmented with Flexiforce™ pressure sensors (Tekscan, Inc., Boston, MA) embedded in silicone in the pads of the thumb, index, and middle fingers. A custom aperture sensor underneath the cosmetic glove encoded the position of the prosthetic hand’s single degree of freedom. The sensor information was sent through a cable to the external nerve stimulator, which converted the sensor information into electrical stimulation pulses. The stimulation traveled to the nerve via percutaneous leads to Flat Interface Nerve Electrodes (FINEs) implanted on the participants’ median nerves. Illustration courtesy of Cleveland FES Center. (**B**) Image of a subject wearing the sensory restoration system. (**C**) Study timeline. The study was a three-stage crossover design, in which the subjects used the system at home either without sensation (stages 1 and 3) or with sensation (stage 2). Functional metrics were administered in laboratory testing sessions at the start of the study and after each stage. (**D**) Study stage durations for subject 1 (left) and subject 2 (right). Subject 2 wore the system for nearly twice as many days per stage as subject 1. Subject 1 experienced 3 days of interruption per stage due to component breakage (see Methods). (**E**) Locations of sensory percepts associated with each prosthetic hand sensor. Sensation locations reported daily throughout the study were overlaid such that regions of higher opacity were more-frequently reported. (**F**) Stimulation charge delivered to each channel for subject 2 over the course of the sensory-enabled stage of the study. Participants could calibrate stimulation settings (pulse amplitude and pulse width) whenever they chose. Filled dots are stimulation settings at each recalibration; color corresponds to the percept locations in subpanel E. The slopes of the regressions for the thumb, index, and middle channels (navy blue, teal, and magenta) were not significantly different from zero (regression slope test, p > 0.1 for all). The slope for the aperture channel (brown) was −0.11 nC/hr and was significantly less than zero (p = 0.002).

In this study, for the first time, two subjects used a naturalistic sensory restoration prosthesis with direct nerve interfaces autonomously in a home setting (Fig. 1). Sensors embedded on a single degree of freedom (DOF) myoelectric hand controlled electrical stimulation that was delivered to the subjects’ median nerves via implanted Flat Interface Nerve Electrodes (FINEs)^21,23^. The participants felt tactile sensation on their thumb, index, and middle fingers corresponding to the appropriate prosthetic fingertip sensor, and the sensations were independently modulated for intensity. They also felt either proprioceptive sensation or a substituted tactile sensation corresponding to the aperture of the prosthetic hand. The home study consisted of a three stage quasi-experimental design: During the Pre-test and Post-test stages, the subject wore the provided single DOF hand with standard agonist/antagonist control and embedded sensors daily outside of the lab, but did not receive nerve stimulation for sensation. During the sensation-enabled intervention stage, the subjects wore the same single DOF hand with embedded sensors and received nerve stimulation corresponding to readings from the sensors. Comprehensive data was collected daily throughout the home study and at post-stage testing sessions to evaluate the impact of restored sensation at home (Table 1). We developed a theoretical framework to understand the holistic impact of restored sensation on the experience of having a hand that can feel, defining key domains of prosthesis usage, functional task performance, and psychosocial outcomes. This paper reports on the impact of a sensory restoration system on these three domains in the first study of community use of a prosthesis with sensation provided by a direct nerve interface.

**Table 1:** Study measures and data collection schedule, indicating when measures were administered and how they were analyzed.

			Daily Measures	Measures in Testing Sessions	
			*Longitudinal Comparisons*	*Longitudinal Comparisons*	*Sensation on vs off*
Outcome Measures	Usage	Duration of use	• Daily diary of wear time • Onboard log of wear time		
		Task willingness	• Proportion of modified UEFS tasks performed		
		Active usage	• Pressure sensor data		
	Functional	Dexterity		• Clothespin relocation test • Nine hole peg test	• Clothespin relocation test • Nine hole peg test
		Decision making		• Magnetic table test • Foam block identification test	• Magnetic table test Foam block • identification confidence
		Activity performance		• AM-ULA	• AM-ULA
	Psychosocial	Embodiment	• PEM (short form)	• PEM (long form) • RHI embodiment survey	
		Perception of abilities	• PEM (short form) • Modified UEFS difficulty	• PEM (long form) • PSFS • QuickDASH • Magnetic table test confidence • Foam block identification confidence	• Magnetic table test confidence • Foam block identification confidence
		Social interactions	• PEM (short form)	• PEM (long form)	
		Quality of life		• OPUS Quality of life	
		Body image	• Perceived limb length	• PEM (long form)	
		Prosthesis efficiency	• PEM (short form)	• PEM (long form)	
Analyses			Statistical tests across daily measures by stage	Comparisons by testing session	Statistical tests between sensation on and off

## Results

### Sensation increases prosthetic usage

**Figure 2:**
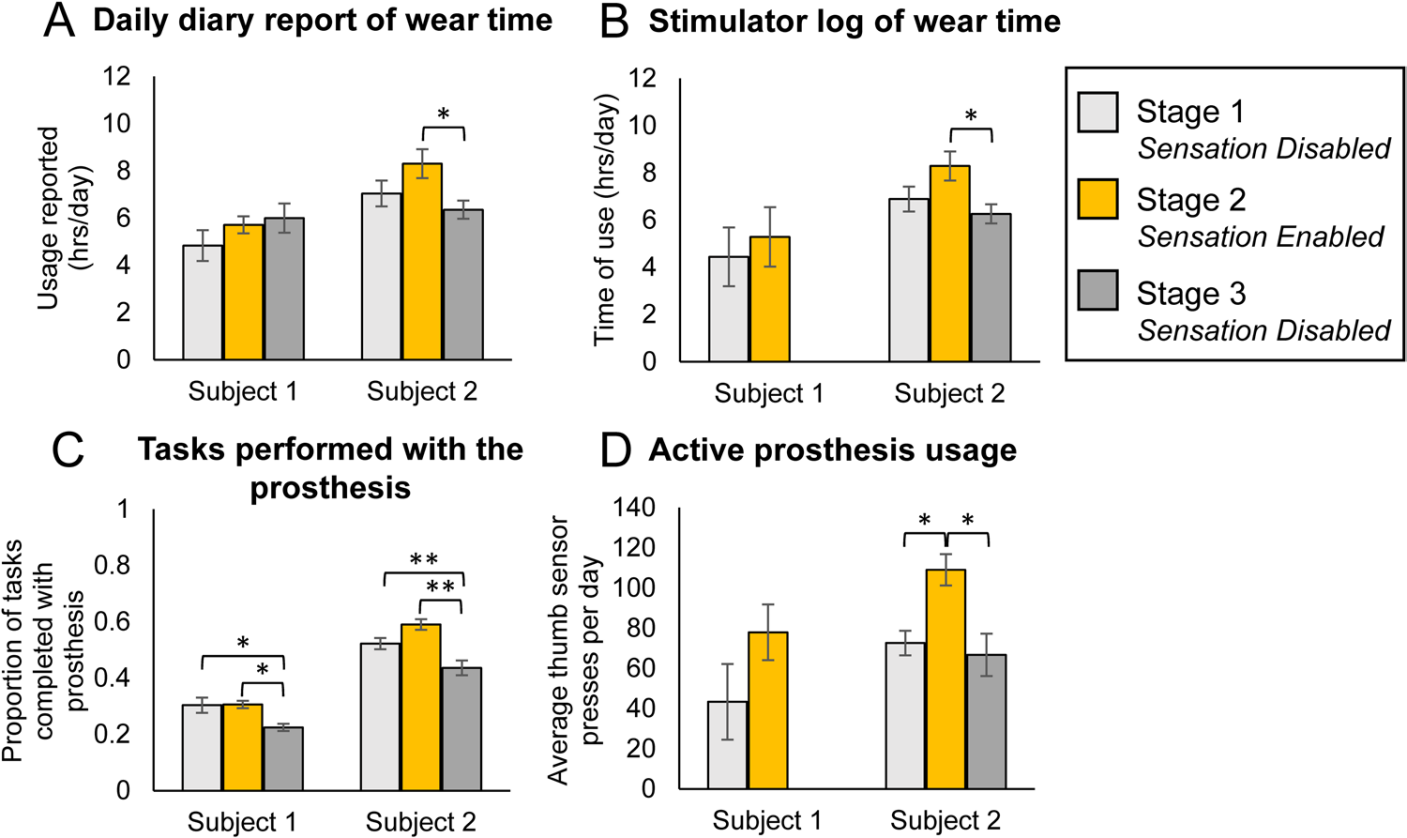
Impact of sensation at home on prosthesis usage. (**A**) Daily diary reports of system wear time (S1: n = 6, 7, 7 for stages 1, 2, 3, respectively; S2: n = 11, 13, 14 for stages 1, 2, 3, respectively). (**B**) Stimulator log of system wear time (S1: n = 5, 4 for stages 1, 2, respectively; S2: n = 12, 13, 15 for stages 1, 2, 3, respectively). (**C**) Tasks performed with the prosthesis each day out of standard task list based on the UEFS (S1: n = 6, 7, 7 for stages 1, 2, 3, respectively; S2: n = 11, 13, 14 for stages 1, 2, 3, respectively). (**D**) Active usage of the prosthesis for grasp-related activities that involved triggering the pressure sensors. Raw sensor data was analyzed for peaks in force, which indicated instances that the prosthesis was used to manipulate or grasp objects (S1: n = 5, 4 for stages 1, 2, respectively; S2: n = 9, 13, 15 for stage 1, 2, 3, respectively). All subpanels: Error bars indicate standard error of the mean. Single asterisk indicates significance of p < 0.05. Double asterisks indicate significance of p ≤ 0.001.

We studied the impact of sensation at home on the participants’ desire to wear and use the system. We monitored wear time using self-reports in the daily diary and an automatic log onboard the system that recorded usage both with and without sensation. Subjects were asked to use the sensory prosthetic for at least 4 hrs per day, but both participants reported wearing the system significantly longer than 4 hrs per day (reported usage combined across all stages: S1: 5.9+/−1.5 hrs/day; S2: 7.2+/−2.0 hrs/day) (one-sample t-test compared to 4 hrs: p < 0.001, both subjects) (Fig. 2A). Subject 1 reported wearing the system longer per day in stage 2 than in stage 1 (p = 0.461). Subject 2 reported wearing the system longer per day when sensation was enabled than when it was not, and this difference was significant between stages 2 and 3 (p = 0.030) (Fig. 2A). The stimulator logs demonstrated objectively that both subjects wore the system for more hours per day when sensation was enabled than when it was not, but this difference was only significant for subject 2 and between stages 2 and 3 (S1: p = 0.141, S2: p = 0.032) (Fig. 2B). Usage duration was not significantly different between the diary reports and the stimulator logs for either subject (paired t-test, S1: p = 0.469, S2: p = 0.379).

In addition to wear time, we examined whether enabling sensation would change *how* the prosthesis was used. We determined the subjects’ willingness to use the prosthesis to do tasks by measuring the proportion of tasks (out of the 28 tasks on the modified UEFS, see Methods) that participants chose to complete each day with the prosthesis. Both subjects performed a significantly greater proportion of tasks when sensation was enabled than when it was disabled in stage 3 (S1: p = 0.007, S2: p < 0.001) (Fig. 2C). The proportion of tasks performed was also significantly less in stage 3 than in stage 1 for both subjects.

Finally, we examined the extent to which the prosthesis was actively vs passively used by comparing the activity on the pressure sensors across stages. If the prosthesis was used more actively, such as in touching or grasping objects, there would be a greater number of peaks recorded in the sensor data, where each peak corresponds to a discrete contact event between the sensor and an object. In contrast, if the prosthesis was infrequently used to grasp or contact objects, we would see fewer peaks in the sensor data.

Indeed, both subjects used their prostheses to touch or grasp objects more frequently when sensation was enabled than when it was not. Because there were differences in activity across the three fingertip sensors, we analyzed each sensor separately. Subject 1 had more presses on all three sensors when sensation was enabled than when it was not, but this difference was not significant for any sensor (thumb: p = 0.201, index: p = 0.375, middle: p = 0.072) (Fig. 2D, Fig. S3). For the thumb and middle sensors, subject 2 had significantly more sensor presses when sensation was enabled compared to when it was not (thumb: p = 0.004, middle: p = 0.001) (Fig. 2D, Fig. S3). Subject 2 also had more presses on the index sensor when sensation was enabled than when it was not, but this difference was not significant (p = 0.108) (Fig. S3). Note that for both the stimulator log of wear time and active prosthesis usage, only the data from stage 1 and part of stage 2 were available for analysis in subject 1 due to a malfunction of the data logger during stage 2.

### Functional performance with sensation is better than without sensation

The functional impact of restored sensation was assessed through standardized functional tasks performed in laboratory sessions at the start of the study and after each study stage (Fig. 1C, Table 1). Each task was performed both with and without sensory feedback in each session. Functional measures were divided into three categories based on content: dexterity measures, decision making measures, and activity performance measures. The clothespin relocation task and nine-hole peg task are standard dexterity measures utilized to study rehabilitation outcomes for a variety of upper extremity musculoskeletal disorders. The magnetic table test and the foam block identification test are tasks that require judgment and decision making based on conscious perception of various sensory inputs. The AM-ULA measures performance of a set of daily tasks based on speed, quality of movements, compensatory movements, and skillfulness of prosthetic use (see Methods for references and complete description of each test).

**Figure 3:**
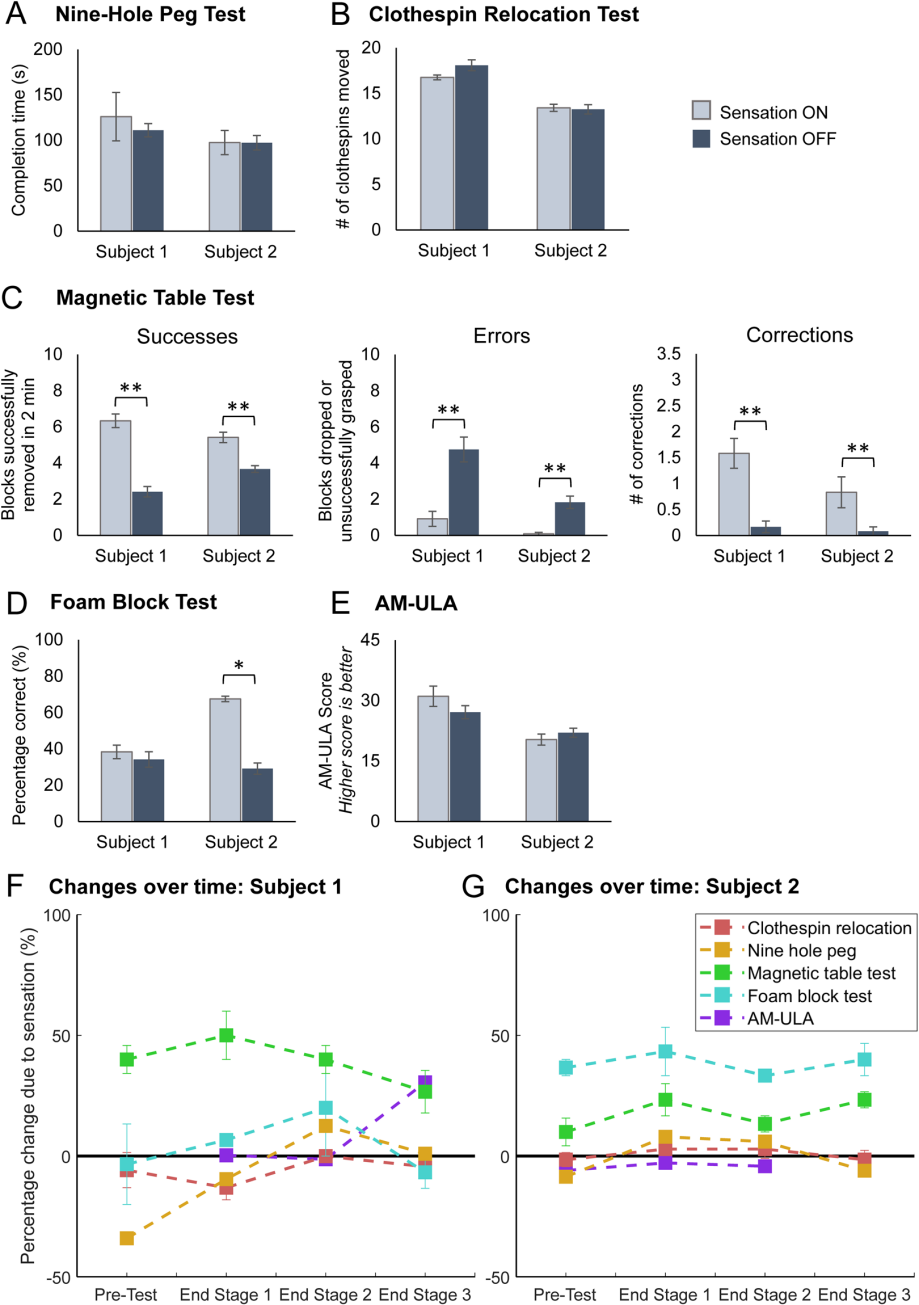
Impact of sensation on functional performance within sessions (**A**–**E**) and over time (**F**,**G**). (**A**) Impact of sensation on the nine hole peg test. Faster completion times indicate better performance (n = 4 for each condition (sensation on/off)). (**B**) Impact of sensation on the clothespin relocation task. More clothespins moved indicates better performance (n = 12 for each condition). (**C**) Impact of sensation on the magnetic table test. Greater numbers of blocks removed in the allotted time (2 min) indicates better performance. Fewer grasping errors and more corrections also indicate better performance (all metrics: n = 12 for each condition). (**D**) Impact of sensation on the foam block identification test. Greater percentages of blocks identified correctly indicate better performance (n = 8 for each condition). (**E**) Impact of sensation on the AM-ULA. Higher scores indicate better performance (The AM-ULA was only scored for 3 of 4 testing sessions for each subject (see Methods); n = 3 for each condition). Panels A–E: Two sample t tests were used to compare performance with and without sensation. Single asterisk indicates significance of p ≤ 0.05. Double asterisks indicate significance of p ≤ 0.001. (**F**) Changes in sensation-enabled functional performance over time for Subject 1 and (**G**) Subject 2. Data is pooled within sessions (n = 3 for each testing session for the clothespin relocation test and magnetic table test; n = 2 for the foam block test; n = 1 for the nine hole peg test and AM-ULA). For **F**,**G**, percentage change due to sensation was calculated as the difference between performance with sensation on and off, normalized by the maximum possible score on the metric (magnetic table test: maximum of 10 blocks; foam block test: maximum of 100% accuracy; AM-ULA: maximum score of 40). The nine hole peg and clothespin relocation test scores were normalized to the maximum score reported across all testing sessions and subjects (see Methods). Points above the black line at zero indicate that performance was better with sensation than without. Points below the black line indicate that performance was worse with sensation than without. All panels: Error bars indicate the standard error of the mean.

Both subjects performed the same on the dexterity tasks when they had sensory feedback as when they did not (clothespin relocation: S1: p = 0.127, S2: 0.691; nine hole peg: S1: p = 0.607, S2: p = 0.785) (Fig. 3A,B). In contrast, the subjects performed better on the decision-making tasks when they had sensory feedback compared to when they did not (Fig. 3C,D). For the magnetic table test, both subjects successfully removed significantly more blocks from the table, had significantly fewer grasping errors, and had significantly more successful corrections of potential errors when they had sensory feedback compared to when they did not (p < 0.001 for all outcomes for both subjects) (Fig. 3C). For the foam block test, subject 2 performed significantly better with sensation than without (S1: p = 0.564, S2: p = 0.002) (Fig. 3D). For the AM-ULA, neither subject performed statistically significantly better with sensation compared to without (S1: p = 0.279, S2: p = 0.407) (Fig. 3E). However, the improvement of subject 1’s performance with sensation compared to without in the last testing session (improvement of 12.2 points) was greater than the size of the minimal detectable change (MDC) for this test, suggesting that the change that occurred was greater than measurement error (MDC-90 = 3.7^27^) (Fig. S1G).

### Home use with sensation may yield additional benefits in sensation-enabled functional performance

We also examined the impact of *home use* of sensation on functional performance by making comparisons across the four testing sessions of this study. These longitudinal comparisons may indicate the degree to which learning occurred. Learning how to operate the new myoelectric hand, the dynamics of the custom silicone fingertips, and/or optimized strategies to perform the tests would appear as improvements over time in performance both with and without sensation. However, learning how to interpret or use the sensory information following extended exposure to sensation at home would appear only in the sensation-enabled performance on the measures. To assess the changes in sensation-enabled performance due to home use, the percentage change in outcome due to sensation was calculated for each testing session and then compared across testing sessions (Fig. 3F,G, see Fig. S1 for raw data). Note that statistical comparisons were not possible because of the low numbers of observations.

Both subjects performed better with sensation compared to without in all testing sessions on the magnetic table test, and subject 2 performed better with sensation compared to without in all testing sessions on the foam block test (Fig. 3F,G, data points above the line at zero). On the magnetic table test, performance ranged from 25–50% better with sensation compared to without for subject 1 and 10–23% for subject 2. On the foam block test, performance ranged from 33–43% better with sensation compared to without for subject 2. On these tests, although performance was always better with sensation compared to without, no additional improvements in performance with sensation were made after using the sensation at home.

However, when performance was initially worse with sensation compared to without (as evidenced by data points below the line at zero in Fig. 3F,G), home use of sensation tended to lead to improvements in sensation-enabled performance. Subject 1’s performance with sensation was better after using the sensation at home than in the other three testing sessions for the foam block test and nine-hole peg test (Fig. 3F). For both of these tests, the subject initially performed worse with sensation compared to without in the pre-test session. However, after using the sensation at home (end stage 2 session), subject 1 performed about 20% better with sensation compared to without on the foam block test and 12% better with sensation compared to without on the nine hole peg test. Interestingly, these performance gains disappeared after again using the system without sensation in stage 3. A similar trend was observed for the clothespin relocation test, where sensation-enabled performance improved after using the sensation at home, but the with-sensation performance never exceeded the without-sensation performance. On the AM-ULA, subject 1 performed about 30% better with sensation compared to without in the final testing session, but performed the same with and without sensation in prior testing sessions.

For subject 2, changes in sensation-enabled performance occurred across sessions for the clothespin relocation test and nine hole peg test (Fig. 3G). For these tests, performance was initially worse with sensation compared to without, but improved after stage 1 such that performance was 3–8% better with sensation compared to without. These performance improvements were sustained through the end stage 2 session, then disappeared after stage 3. There were no changes over time on the AM-ULA for subject 2.

### Home use with sensation improves user experience

**Figure 4:**
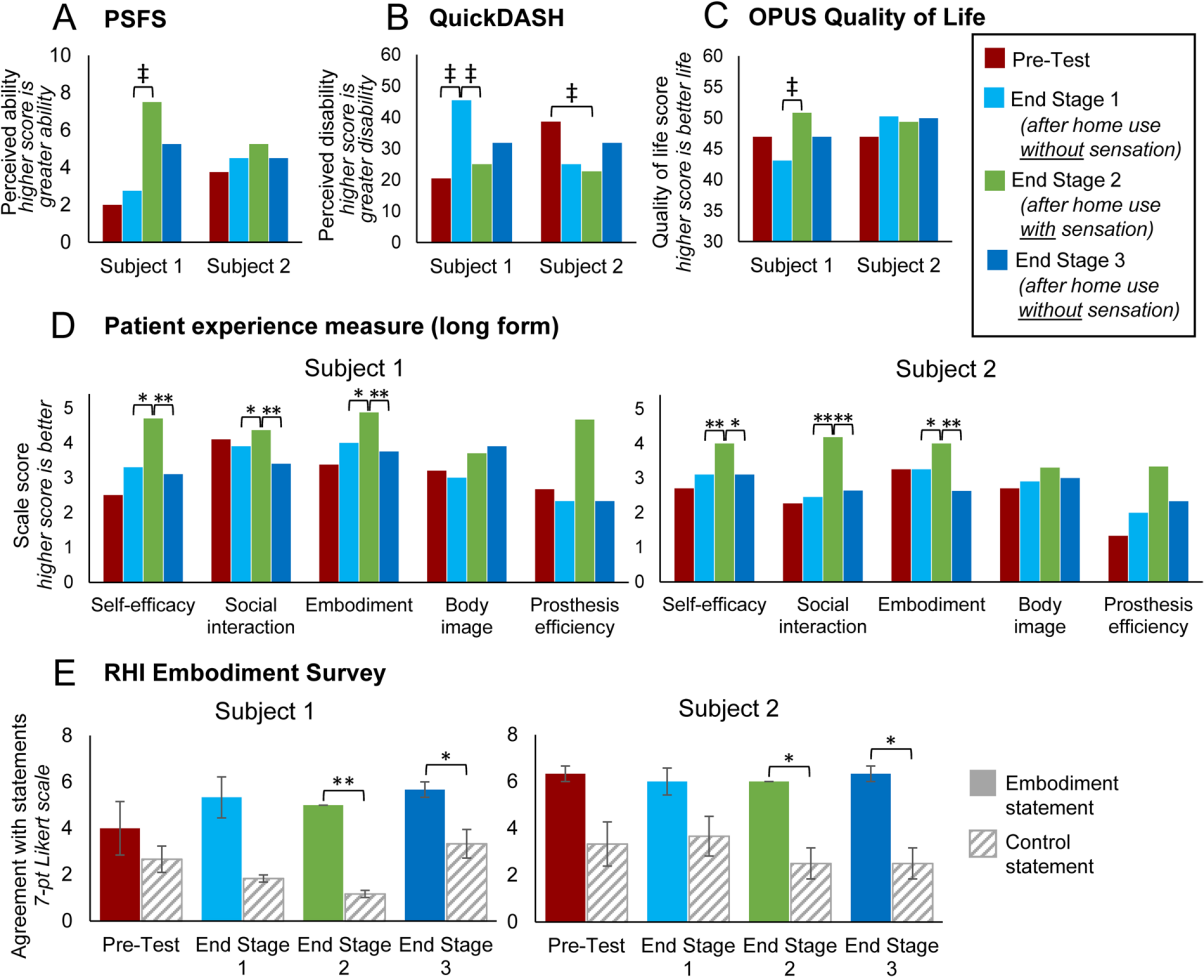
Impact of home use with sensation on end of stage psychosocial measures. (**A**) Patient-specific functional scale (PSFS) scores over time. Higher scores indicate greater perceived ability. (**B**) QuickDASH disability score over time. Higher scores indicate higher perceived disability. (**C**) OPUS Quality of life score over time. Higher scores indicate better perceived life. (**D**) Patient experience scales (long form) scores over time. Scale scores were computed by averaging ratings on items within each scale. Higher ratings indicate better outcomes. Statistical comparisons were made between individual items scores within each scale using paired t-tests. (**E**) RHI embodiment survey. Agreement ratings on the embodiment-related statements are shown as filled bars. Ratings on control statements are shown as striped bars. Embodiment-related statements were compared to control statements with two-sample t-tests. Error bars indicate standard error of the mean. All panels: Each subject completed each survey once per testing session. Single asterisk indicates significance of p < 0.05. Double asterisks indicate significance of p ≤ 0.001. Double cross indicates change greater than the minimal detectable change (MDC-90) reported for that test.

**Figure 5:**
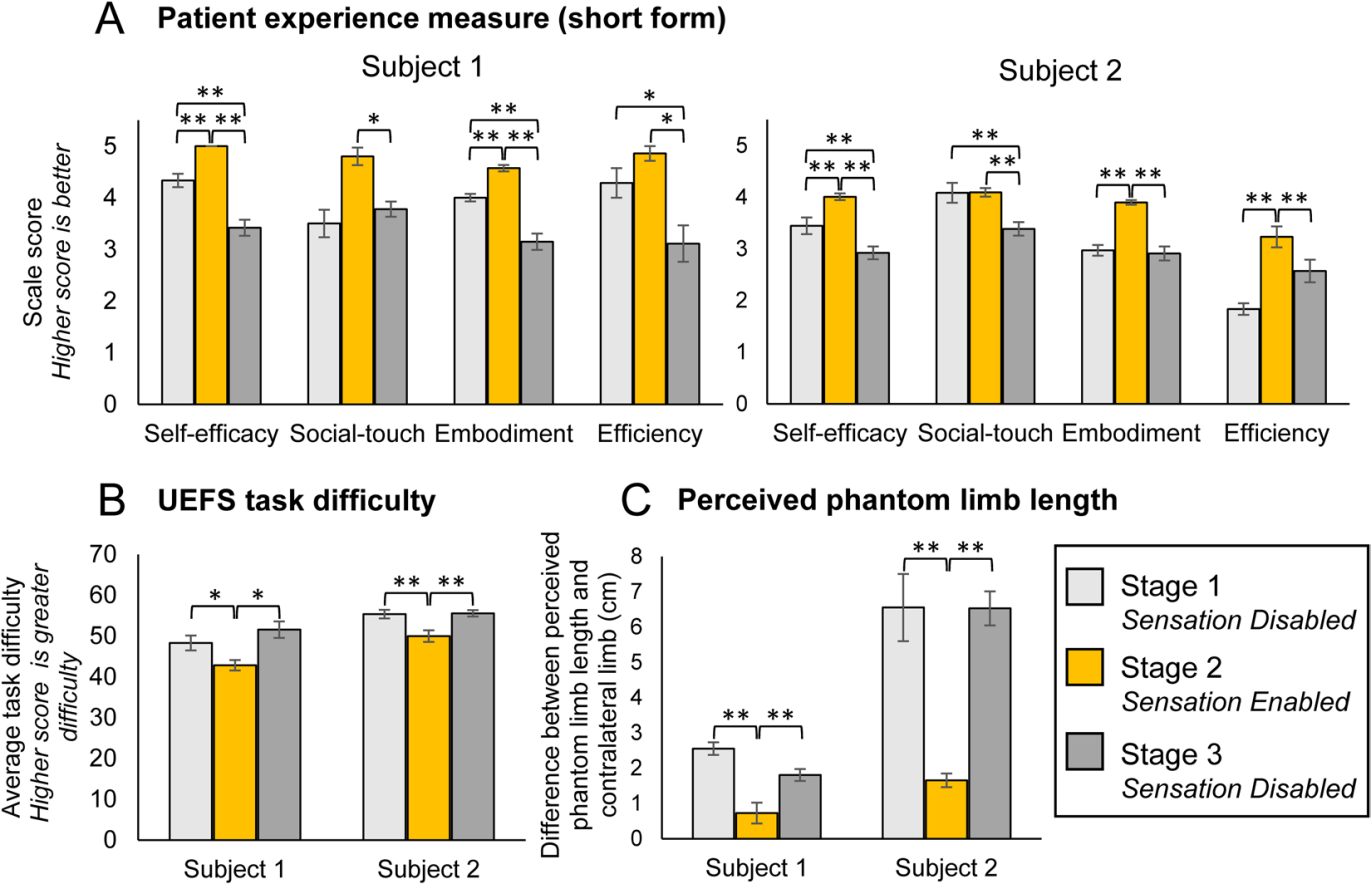
Impact of sensation on daily psychosocial measures. (**A**) Patient experience scale (short form) scores by study stage. Higher scale scores indicate better perceived outcomes in each domain (S1: n = 6, 7, 7 for stages 1, 2, 3, respectively; S2: n = 11, 13, 14 for stages 1, 2, 3, respectively). (**B**) Reported difficulty in task performance by study stage. Each day, participants rated their perceived difficulty in doing each of 28 tasks on a list based on the OPUS UEFS. Higher scores indicate greater perceived difficulty (S1: n = 6, 7, 7 for stages 1, 2, 3, respectively; S2: n = 11, 13, 14 for stages 1, 2, 3, respectively). (**C**) Perceived phantom limb length by study stage. The difference between the perceived phantom limb length and the contralateral limb length indicates the degree of limb telescoping experienced. Lower values of limb length difference correspond to a more natural phantom limb length and thus less disturbance in body image (S1: n = 6, 7, 8 for stages 1, 2, 3, respectively (n = 8 for stage 3 because we included one report from a day of interruption); S2: n = 11, 13, 15 for stages 1, 2, 3, respectively). Note that subject 1’s residual limb is ~20 cm shorter than his contralateral side, while subject 2’s residual limb is ~35 cm shorter than his contralateral side. All panels: Error bars indicate standard error of the mean. One-way ANOVAs with Tukey pairwise comparisons were used to compare outcomes across stages. Single asterisk indicate significance of p < 0.05. Double asterisks indicate significance of p ≤ 0.001.

To assess the impact of sensory restoration on user experience and psychosocial outcomes, we administered multiple surveys to participants both daily throughout the study and at the end of stage testing sessions (Table 1). We then made comparisons over time, either between testing sessions (Fig. 4) or by stage (Fig. 5), depending on the collection schedule of the survey. Whenever possible, we utilized existing surveys reported in the literature, especially those with documented reliability, validity, responsiveness, and minimal detectable change (MDC), so that we could make comparisons to other studies. However, because the existing surveys did not measure many of the topic areas of interest (see full list of psychosocial categories in Table 1), we developed several novel surveys for this study, such as the patient experience measure (PEM)^28^. We assessed six categories of psychosocial outcomes: perception of abilities, embodiment, social interactions, quality of life, body image, and prosthesis efficiency.

### Perception of abilities

In addition to monitoring the impact of home use on subjects’ *actual* abilities to use the prosthesis to do functional tasks, we wanted to measure their *perception* of their abilities. During their home use, the subjects may have done hundreds of tasks with the sensory-enabled prosthesis, and the sum total of these experiences informs their feelings of confidence and self-efficacy, regardless of their performance on standardized tests in the testing sessions. We assessed subjects’ perception of their abilities using several existing measures: the Patient-Specific Functional Scale (PSFS), QuickDASH, and Orthotics Prosthetics User Survey (OPUS) Upper Extremity Functional Status (UEFS). We also assessed confidence during in-lab functional testing and via the PEM.

The PSFS measures the subjects’ perceived ability to do tasks that they self-identify and report are currently difficult to perform (see Methods). This metric is advantageous because it measures perceived ability for tasks relevant to subjects’ lives and interests, but outcomes depend largely on what specific tasks each subject chose to evaluate. Subject 1 identified the tasks of grabbing fruit, grabbing fragile items, touching people, and performing precise movements. Subject 2 identified the tasks of retrieving items from a shelf, cutting meat, riding a bicycle, and peeling potatoes. Subject 1 was significantly impacted by having sensation at home, because his PSFS score increased above the MDC between the end stage 1 and end stage 2 in-lab sessions (MDC-90 = 2.34 points^29^) (Fig. 4A). His score also decreased substantially (2.25 points) between the end stage 2 and end stage 3 in-lab sessions, indicating that his perceived ability to do the identified tasks decreased when he no longer had sensation at home. Subject 2’s PSFS score was highest after having sensation at home, but the score differences were not above the MDC.

The Quick-DASH is a standard measure of perceived disability (see Methods). Subject 1’s perceived disability score increased more than the MDC (MDC-90 = 13.9^30^) after using the system at home without sensation, and decreased more than the MDC after using the system at home with sensation (Fig. 4B). Subject 2’s perceived disability score decreased more than the MDC between the pre-test and end of stage 2 in-lab sessions (15.9 points), but not compared to the end of stage 1 session. The end of stage 3 scores increased above the end of stage 2 scores for both subjects, but these changes were not above the MDC.

We investigated participants’ perception of their abilities in relation to specific functional tests. Before every trial of the functional tasks in the decision making category (magnetic table test and foam block identification test), the subjects were asked to rate their confidence in their abilities by predicting what their performance in the upcoming trial would be. For both tasks, both subjects were significantly more confident when sensation would be enabled in the upcoming trial than when it would be disabled (p < 0.001, both task confidence ratings, both subjects) (Fig. S2A,B). For both task confidence ratings, there was no impact due to testing session (magnetic table test: S1: p = 0.801, S2: p = 0.060; foam block test: p = 0.063 both subjects).

We also assessed participants’ perception of their self-efficacy using the self-efficacy subscale of our novel PEM metric. On the self-efficacy scale of the PEM long-form, both subjects reported significantly higher scores after having sensation at home than in any other session, reflecting greater confidence in their abilities to use the prosthesis when they have sensation (end stage 1 vs end of stage 2: S1: p = 0.02, S2: p = 0.001; end stage 2 vs end of stage 3: S1: p < 0.001, S2: p = 0.004) (Fig. 4D). On the self-efficacy scale of the PEM short-form, self-efficacy scale scores were significantly higher when sensation was enabled than in either of the sensation disabled stages, indicating significantly more confidence with sensation (p < 0.001, both subjects) (Fig. 5A). Both participants also reported significantly higher self-efficacy scores in stage 1 than in stage 3 (p < 0.001, both subjects).

Finally, in our modified version of the OPUS UEFS, subjects rated the difficulty of 28 common tasks daily (such as brushing teeth, putting on socks, using a key in a lock, and using a fork or spoon; see Methods). Both subjects reported that tasks were significantly easier (lower difficulty score) when sensation was enabled than when it was disabled (S1: p = 0.007, S2: p < 0.001) (Fig. 5B).

### Embodiment

We asked whether using the prosthesis with sensation would yield incorporation of the prosthesis into the body schema (embodiment). Participants completed the rubber hand illusion (RHI) embodiment survey immediately after performing an in-lab functional task with sensation enabled. The RHI survey determines whether a prosthesis is or is not embodied, by comparing subjects’ ratings on statements related to embodiment to control statements (see Methods). Although agreement with the embodiment questions was always greater than the control questions, this difference was only significant for the two testing sessions following the sensation enabled stage of the study (pre-test: S1: p = 0.539, S2: p = 0.053; end of stage 1: p > 0.1, both subjects; end of stage 2: p < 0.005, both subjects; end of stage 3: p < 0.05, both subjects) (Fig. 4E). Although subjects experienced embodiment after performing in-lab functional tests with sensation, they only experienced significant prosthetic embodiment after using the prosthesis with sensation at home.

On the PEM embodiment scale long-form, both subjects had significantly higher scale scores, reflecting greater embodiment of their prostheses, after stage 2 of the study, which was the sensation-enabled stage (end stage 1 vs end of stage 2: p < 0.02, both subjects; end stage 2 vs end of stage 3: p < 0.001, both subjects) (Fig. 4D). On the PEM embodiment scale short-form, both participants reported significantly higher scale scores, indicating significantly more embodiment, when sensation was enabled than in either of the sensation disabled stages (p < 0.001, both subjects) (Fig. 5A). For subject 1, embodiment scores were significantly greater in stage 1 than in stage 3 (p < 0.001).

### Social Interaction

We developed the PEM social interaction scale to assess an amputee’s perception of their ability to use their prosthesis in social greetings and to communicate or connect with others. On the PEM social interaction scale long-form, both subjects had significantly higher scale scores, reflecting better perceived social interaction, after having sensation at home (end of stage 1 vs end of stage 2: S1: p < 0.05, S2: p < 0.001; end of stage 2 vs end of stage 3: p ≤ 0.001, both subjects) (Fig. 4D). On the PEM short-form, both subjects reported significantly higher perception of their social touch abilities during stage 2 than in stage 3 (S1: p = 0.002, S2: p < 0.001), but stages 2 and 1 were indistinguishable (Fig. 5A).

### Quality of Life

We assessed the impact of home use with sensation on participants’ overall quality of life using the OPUS Quality of Life index (see Methods). The individual item ratings are combined into an index score using a keyform where higher scores indicate a better quality of life. Subject 1 reported an increase in quality of life between the end of stage 1 and the end of stage 2 sessions that was above the MDC (MDC-90 = 7.4^31^) (Fig. 4C). No other changes exceeded the MDC.

### Body Image

We developed the PEM body image scale to understand how having an amputation, specifically when the prosthesis is removed, impacts one’s perception of self and perception of how others view oneself. On the PEM body image scale long-form, Subject 2 reported higher scale scores after the sensation enabled stage, reflecting improved perception of self (Fig. 4D). Subject 1’s score steadily rose throughout the study and were highest at the end of stage 3. However, no inter-session comparisons were significant. There was no short form for this scale.

In addition to assessing holistic body image, we were interested in whether sensory feedback would change the participants’ view of their amputated limb, specifically their experience of limb telescoping. Many amputees experience limb telescoping, a phenomenon in which the phantom hand retracts proximally such that it is perceived to be collocated with the residual limb^32^. To assess perceived phantom limb length, participants drew a line on an image of an intact arm where they perceived the most distal location of their phantom fingertips. The distance between the drawn line and the fingertips of the image represents the discrepancy between the perceived phantom limb length and the contralateral limb length (the “true” limb length). Both subjects perceived that their limbs were significantly longer (closer to contralateral length) during the sensation enabled stage than in either of the sensation disabled stages (p < 0.001, both subjects) (Fig. 5C).

### Prosthesis Efficiency

We developed the PEM prosthesis efficiency scale to assess participants’ perception of the focus required to use their prosthesis and speed of using their prosthesis. On the PEM long-form, both participants rated their prosthesis efficiency to be substantially higher after the sensation enabled stage of the study than in any other testing session, but these differences were not significant (end stage 1 vs end of stage 2: S1: p = 0.07, S2: p = 0.18; end of stage 2 vs end of stage 3: S1: p = 0.07, S2: p = 0.23) (Fig. 4D). On the PEM short-form, subject 1 had significantly higher perceived efficiency during stage 2 than in stage 3 (p = 0.003), but ratings were indistinguishable between stages 1 and 2 (Fig. 5A). Subject 2 had significantly higher perceived efficiency during stage 2 than in either of the other stages (p < 0.001).

## Discussion

This is the first study in which multi-channel sensory stimulation was used autonomously by prosthesis users in home and community settings without daily researcher supervision. Participants could use the sensory-enabled prosthesis the way they would an intact hand: they described using the system to do chores, shake hands with friends, carry their morning coffee, and play with their grandchildren. The participants were able to experience, for the first time, how sensory restoration was applicable and valuable to their daily lives. Both participants were overwhelmingly positive about the experience and stated clear preferences for using the prosthesis with sensation rather than without. One participant stated that he wore his prosthesis longer than normal because of the sensory restoration, explaining “That’s because I like the sensation of having my hand there, and feeling like my hand was there, so I didn’t want to take it off.” When approaching the end of the sensory-enabled stage of the study, one participant expressed his feelings about losing the sensation: “Tonight’s my last night with it, when I come back, I gotta wear it another 3 weeks, but that’ll be without sensation. That’s like losing your hand all over again!”.

We developed a framework to understand the holistic impact of having a hand that can feel by categorizing our outcome metrics into three domains. Our data demonstrates that sensation resulted in improvements in metrics of user experience, prosthesis usage, and functional performance. We also show that daily use of a sensory-enabled prosthesis resulted in improvements in sensation-enabled functional performance for subject 1, which may be due to learning.

The largest and most consistent effects of sensory restoration were the improvements in psychosocial self-rated measures. Overall, home use with sensation positively improved the experience of using the prosthesis. Both participants reported a lower severity of their perceived disability, improved perception of their ability to use the prosthesis to do tasks, and a normalization of their body schema, as evidenced by a more natural phantom limb length. On the PEM, participants reported greater embodiment of their prostheses, improved perception of their ability to use the prosthesis to interact with others, higher confidence, and enhanced perception of prosthesis efficiency. Subject 1 reported that sensation enabled an overall better quality of life. While we, and others, have demonstrated that in-lab-use of sensation can impact prosthetic embodiment and self-confidence^18,25^, this is the first evidence that sensory feedback can significantly impact perceived disability, perception of social abilities, prosthesis efficiency, phantom limb length, and quality of life. Importantly, these improvements in user experience occurred after mere days of home use with sensation and without any change to the prosthesis’ appearance, dexterity, or fit. We are interested to see in future studies whether longer durations of home use with sensation and/or the combination of sensation with more dexterous prostheses or advanced control strategies would result in even larger effects. The improvements in user experience demonstrated here show that sensory feedback fundamentally reshapes the way that prosthetics users view their prosthesis in relation to their bodies, their abilities, and their interaction with others.

Sensory feedback increased the subjects’ willingness to wear the prosthesis and willingness to use the prosthesis to do tasks. The increased prosthesis usage attributable to home use of sensation demonstrated in this study provides encouraging evidence that sensory feedback could improve prosthesis adoption and reduce abandonment. The current rate of prosthetic abandonment is 23% for myoelectric prostheses and 28% overall, with 85% of prosthesis rejecters citing the lack of sensory feedback as a factor in their decision not to wear a prosthesis^33,34^. Decreasing abandonment could improve patient outcomes and reduce costs for the healthcare system. Further, upper limb prostheses are typically used in supporting (assisting) or stabilizing roles, with amputees relying heavily on the contralateral limb and/or compensatory movements of the trunk to perform tasks. Consequentially, overuse injuries are common in upper limb amputees^35,36^. This study provides the first evidence that restored sensation could modify the user’s approach to using the prosthesis. More active use of a prosthesis rather than overusing the contralateral limb could lead to reduced compensation injuries, thereby further improving patient quality of life and reducing healthcare costs.

As expected, the addition of sensory feedback improved performance on “decision making” functional tasks - those that involved consciously perceiving the sensory information, when available, and making movements or verbal identifications as appropriate in response to this information^18,25^. When sensory feedback was disabled, the subjects could only use vibrations or other sensations transmitted through the socket to make decisions, since they did not have any visual or auditory feedback. Both participants reported that their primary strategy in the sensation-disabled condition was guessing. When sensory feedback was enabled, the subjects could perceive tactile and proprioceptive sensations associated with object manipulation and make their decisions for the tasks by interpreting this information. The significant improvements with sensation show that the subjects could interpret the sensory information appropriately to make correct decisions about how to perform the tasks.

The addition of sensory feedback did not impact performance on dexterity or activity performance measures. This finding might be explained by the prosthetic hardware (which was consistent across conditions) as well as by the choice of performance metrics. In the dexterity tasks, because they were using a single DOF prosthetic hand, the participants tended to close the hand to a fixed aperture, then use the shoulder to complete the movements, which would not employ the sensory feedback system provided through stimulation. The dexterity tasks also involved grasping rigid, non-breakable objects and moving the objects in space as quickly as possible. Both of these factors – lack of concern for object breakage and preference for speed over precision of grasp – decrease the likelihood that the participants would need or use the sensory information provided by stimulation. Our measure of activity performance, the AM-ULA, utilizes a complex scoring rubric that weights factors including task completion, speed of movement, movement quality and skillfulness of prosthesis use in assigning individual item scores. Given that both subjects had ingrained patterns of compensatory movements of trunk leaning and shoulder substitution (aspects of movement quality) and were not given physical therapy or prosthetic training in this study, their low movement quality ratings influenced scores downward. Thus, without changing the scoring rubric and without training, the AM-ULA was not responsive to other potential changes (in prosthetic skill or speed of performance, for example) that may have resulted from enabling sensation. We also anticipate that the combination of sensory feedback with multi-articulated, dexterous prostheses with advanced control strategies will yield better performance on these tests.

Despite the short duration of the study, subject 1 demonstrated improvements in functional performance with sensation after using sensation at home in some metrics. This suggests that daily experience with sensory feedback for multiple consecutive days promotes learning how to interpret the sensory information and apply it to functional tasks. However, more data is needed to better quantify this process. In addition, we did not see improvements over time on metrics in which subjects already performed much better with sensation compared to without before the sensation-enabled stage of the study, such as on the magnetic table test (for both subject 1 and 2) and the foam block test (for subject 2). This may be because the learning process had already occurred for these tests before the home use study, since the subjects had previously performed hundreds of trials of the foam block test and the magnetic table test for prior studies^18,25^. Following this learning process, a performance ceiling related to the dexterity or control scheme of the prosthesis may have prevented further improvements due to home use of sensation. Interestingly, the performance gains over time observed for some metrics for both subjects 1 and 2 disappeared when sensation was disabled again in stage 3. This trend of decreased outcomes in stage 3 also occurred for nearly all prosthesis usage metrics and psychosocial metrics. In some cases, the only significant differences were between stages 2 and 3. These trends may indicate that sensory restoration shifted the participants’ perspective of their status quo towards greater functionality and connectedness, and that subsequently removing the sensation was viewed as a loss of capabilities. This hypothesis is supported by the subject’s comment, highlighted above, that using the system without sensation was like “losing your hand all over again”. The trends could also reflect increasing levels of frustration with the system over time or a decreased willingness to wear and use it. For subject 1, frustration with the system may have been exacerbated by the breakages and interruptions experienced throughout the study.

The findings of enhanced psychosocial outcomes on the PEM are encouraging, but need to be interpreted cautiously. The PEM is a new measure, developed for our study. The measure has face and content validity, however no data on PEM reliability and validity of the PEM has been reported to date. Future research is needed to obtain normative data on the PEM and obtain additional data to validate the measure. In addition, although the effect of sensory restoration on magnetic table test performance, PEM outcomes, perceived phantom limb length, prosthesis embodiment, and UEFS task difficulty were consistent across subjects, some metrics demonstrated subject-specific differences. The subject-specific differences related to prosthesis usage were largely due to the subjects’ differences in lifestyle and experience with system breakage. Subject 1 was a full time student at the time of this study and experienced several days of interruption due to system component breakage. These factors decreased the time available for him to wear the system, his opportunities to actively use the prosthesis, and the statistical power of our comparisons. In contrast, subject 2 is a homemaker and experienced no breakages or interruptions. Thus, he had fewer restrictions in his schedule and more opportunities to use the prosthesis to do tasks. The differences in the PSFS reflect the participants’ choices for which tasks to evaluate, since subject 1’s tasks were more related to precision grasp control than were subject 2’s (see Methods). The differences in the foam block test may be related to the sensory information provided by the aperture sensor. Subject 1 received a referred tactile sensation associated with the aperture sensor, while subject 2 received a proprioceptive sensation of hand closing. Perhaps the higher congruence of the sensation with the information it provided made it easier to interpret for subject 2. The impact of sensation on activity performance was only observed for subject 1. This may be because subject 2’s AM-ULA scores were dominated by body compensation, which may have occluded any smaller changes due to sensory restoration.

Our ability to successfully implement a take home system demonstrates the stability of our technology. We have now demonstrated that our extraneural cuff interface is stable for over five years^21,22^. In addition, this study shows that our interface can reliably evoke sensory percepts across multiple days, with participants only making small adjustments to the stimulation as desired (Fig. 1D). The sensory percepts themselves were stable in location and intensity throughout the duration of the study (Fig. 1C) and were primarily described as pressure or touch (Table S1). Extraneural cuff stimulation is a feasible method of long-term sensory restoration in community use.

The experience of having a hand is multifaceted, including both actual and perceived functional abilities; the emotional and psychological association with one’s body, lifestyle, and identity; and the ability to communicate and relate socially. Sensation plays a vital role in the experience of having a hand. Restored sensation through peripheral nerve stimulation can improve prosthesis function and help upper limb amputees perceive their prosthesis as a hand. Understanding the contribution of any new prosthetic or neural-interfacing technology to the holistic experience of having a hand again is critical to achieving optimal user outcomes. The ability to convey the experience of the hand through a human-machine interface via artificial sensation expands the horizon of possibilities for persons living with limb loss. Further, human-machine interfaces that incorporate the human element of touch may lead to new frontiers of connectedness for persons with limb loss and able-bodied individuals alike. Artificial sensation through direct nerve interfaces enables people to fully share personal experiences with one another, through the physical and emotional connection of touch.

## Materials and Methods

### Subjects

Two persons with unilateral upper limb amputation participated in this study. S1 had a right transradial amputation in 2009 due to a traumatic injury. In 2012, S1 was implanted with two 8-channel Flat Interface Nerve Electrodes (FINEs) around his median and ulnar nerves and a 4-channel CWRU spiral electrode around his radial nerve. S2 had a transradial amputation approximately 3 inches distal to the elbow in 2004 due to a traumatic injury. S2 was implanted with three 8-channel FINEs around his median, ulnar, and radial nerves in 2013. Both subjects were users of myoelectric prostheses prior to enrollment in the study. Subject 1 had been using a myoelectric prosthetic device regularly for 4 years before the take home study. Subject 2 had been using a myoelectric prosthetic device regularly for 3.5 years before the take home study. Both report using a myoelectric prosthesis as their primary prosthetic device, and wear it most of the day (8–12 hours) (when it isn’t being repaired).

The home use study took place during 41and 42 months post-implant for both subjects. All study devices and procedures were reviewed and approved by the U.S. Food and Drug Administration Investigational Device Exemption, the Cleveland Department of Veterans Affairs Medical Center Institutional Review Board, and the Department of the Navy Human Research Protection Program. All study procedures and experiments were performed in accordance with relevant guidelines and regulations of these institutions. Informed consent was obtained from both subjects.

### Home Use System

The home use system consisted of a sensorized prosthetic hand, an external nerve stimulator, a cable to convey the sensor information to the stimulator, and a cable to convey the sensory stimulation to the subject’s nerves via percutaneous leads (Fig. 1A). The sensorized hand was an Ottobock VariPlus Speed™ prosthetic hand (Ottobock, Vienna, Austria) embedded with Flexiforce™ pressure sensors (Tekscan, Inc., Boston, MA) on the tips of the index, thumb, and middle finger and a custom aperture sensor to measure the span of the prosthetic opening. These modifications were made in-house. The stimulator was an external, programmable unit that delivered current-controlled, biphasic, cathodic-first stimulation pulse trains to the FINEs through percutaneous leads. A participant wearing the full system is shown in Fig. 1B. Pressure on a fingertip sensor and the span of the prosthesis grip caused stimulation to be delivered to individual FINE contacts, yielding perceived sensation on the subject’s missing hand (Fig. 1E). The amount of pressure applied, in the case of the fingertip sensors, or degree of prosthetic opening, in the case of the aperture sensor, were conveyed by scaling the stimulation pulse frequency. Both subjects wore their own prosthetic sockets for the study and both have standard agonist/antagonist control using surface EMG.

### Study Design

The home use study was a three stage quasi-experimental design. In Stages 1 and 3, the subjects wore the system without sensory feedback. In Stage 2, the subjects wore the system with sensory feedback (Fig. 1C). After initial in-lab training with the system, the subjects were given autonomous use of the sensory feedback system in their homes and communities. They donned and doffed the system independently, charged their systems, made all cabling connections, calibrated their stimulation levels on a per-location basis, and calibrated the pressure and aperture sensors on the prosthetic hand. Subjects were asked to wear the system for at least four hours a day, but could choose when and how long to wear the system or to wear their personal prosthesis or no prosthesis at any time.

For subject 1, each stage was 12 days in duration. Subject 1 reported wearing the system for 7 days in each stage and chose not to wear the system for 2 days in each stage (Fig. 1D). This was the first use out of the lab and several cabling and hardware issues needed to be resolved, which reduced the number of days he was able to wear the system. A problem with the stimulator recharger in stage 1 required him to return the stimulator to the lab for 3 days for repair. In stage 2, a problem with the stimulator software and a mechanical issue with a cable required him to return the system to the lab for repair for 3 days. In stage 3, the middle finger of the prosthetic hand became mechanically unstable due to the aperture sensor modifications to the hand, which prevented the subject from wearing the system for 3 days until a repair was made. In addition, a failure of the onboard data logger in the middle of stage 2 prevented collection of wear time and sensor data for half of stage 2 and all of stage 3.

For subject 2, stages 1 and 2 were 13 days in duration and stage 3 was 21 days in duration (Fig. 1D). The subject reported wearing the system for 12 days, 13 days, and 15 days in stages 1, 2, and 3, respectively. After solving the technical issues experienced by Subject 1, Subject 2 experienced no major hardware failures and no days of interruption in system usage. For testing sessions, subject 2 returned to the lab within each scheduled week, with the specific testing days based on his availability. However, an illness in stage 3 prevented him from returning to lab as scheduled, extending his stage 3 to three weeks. The subject chose not to wear the system for 1 day in stage 1 and 6 days in stage 3. The subject wore the system every day in stage 2.

### Data Collection Schedule

At the start of the study and after each stage, the subjects visited the lab for two 6-hour in-lab testing sessions. In these testing sessions, several surveys and functional tests were administered and/or graded by researchers. There were a total of four testing sessions (Pre-Test, End of Stage 1, End of Stage 2, and End of Stage 3) (Fig. 1C). The functional measures and surveys associated with functional measures were completed with both sensation on and off during each testing session. Throughout the duration of the study (Stages 1, 2, and 3), the subjects filled out daily diary forms that included survey questions about their experiences, ratings of tasks performed with their prostheses, and free response sections to describe any problems, experiences, or circumstances of note. During Stage 2, when sensation was enabled, the subjects also filled out surveys regarding the location, intensity, and modality of the evoked sensations from sensory stimulation. Each day’s surveys were placed in an opaque, sealed envelope and the subjects were instructed not to reopen the envelopes once closed. Daily surveys completed on testing session days were not included in the analyses. Usage data for testing session days were also not included in the analyses.

### Outcome Measures

This study used a battery of tests and surveys to measure function, user experiences, and prosthesis usage, areas our team believed were key outcomes to demonstrate efficacy. Several sub-domains within these broader categories were also identified (Table 1). Existing metrics that could measure these domains and subdomains were utilized when possible. In some cases, existing metrics were modified to more specifically apply to this study. When existing metrics could not be identified or modified, novel metrics were developed.

### Prosthesis Usage Measures

#### Daily diary

Upon doffing the system at the end of each day, study participants self-reported in their daily diary how many hours they wore the system that day.

#### Onboard usage logs

An electronic activity log built into the stimulator recorded the timestamps of button presses by the user. For example, study participants could use the buttons on the stimulator enclosure to change stimulation settings and calibrate their prosthetic sensors. In addition, the onboard logs recorded the timestamps of when the stimulator was turned on and off. These timestamps were used to objectively quantify the number of hours that the participants wore the system per day.

#### Orthotics Prosthetics Users Survey (OPUS) Upper Extremity Functional Status (UEFS) Proportion of Tasks Completed

Each day, participants indicated how many tasks out of a list of 28 common tasks they completed with the sensorized prosthesis^37,38^. We utilized a modified version of the OPUS Upper Extremity Functional Scale (UEFS)^37^, a measure of everyday activity performance for use with upper limb adult amputees. The 28 items included the original items, and additional items as tested by Jarl (peel potatoes (or fruit) with a knife/peeler, open a bag of chips, take banknote out of the wallet, twist a lid off a small drink bottle, and sharpen a pencil) were added to the new version^38^.

#### Sensor presses

The pressure sensors built into the prosthetic hand streamed data to the stimulator for real-time intensity modulation of the sensory stimulation and was simultaneously recorded at 100 Hz. The raw data was processed offline after study conclusion using a custom script written in Matlab. The script automatically identified the peaks in each sensor’s raw data, representing peaks in pressure on each fingertip sensor. The magnitude and timestamp of each peak was recorded. The total number of peaks for each day was calculated and taken as a measure of the number of prosthetic finger presses on that fingertip.

### Functional measures

#### Clothespin relocation task

For this metric, the subject was asked to remove as many clothespins from a horizontal bar and place on to a vertical bar as possible within 2 minutes^39–41^. This test was repeated three times under three different cognitive load conditions. In condition 1, the subject was asked to move the clothespins while simultaneously counting down from 100 by 3’s. In condition 2 the subject was asked to move the clothespins while simultaneously naming fruits and vegetables. In condition 3, the subject moved the clothespins without performing another cognitive task.

#### Nine-hole peg test

The nine hole peg test is a clinical measure commonly used to assess hand and arm function following stroke^42–49^. In this test, the subject was asked to pick up small pegs individually from a dish and place each into a small hole in a flat board. Once all pegs had been placed, the subject was asked to remove all the pegs and place them back into a dish. The outcome is the duration of time the subject took to complete the test.

#### Activities Measure for Upper Limb Amputees (AM-ULA)

In the AM-ULA, participants completed 18 tasks representing everyday functional activities, such as putting on a t-shirt, zipping a jacket, tying shoelaces, using a fork, drinking from a cup, and folding a towel. The AM-ULA was developed to assess adults with upper limb amputation and considers task completion, speed, movement quality, skillfulness of prosthetic use, and independence in its rating system^27^. All sessions were videotaped and each was assigned a random identification code. The video (without audio) was reviewed and the tests scored by author L.R., who was blinded to the coding scheme. The MDC-90 for the AM-ULA is 3.7 points^27^. For subject 1, the AM-ULA was administered in the end of stage 1, end of stage 2, and end of stage 3 testing sessions, but not in the pre-test session due to limitations in experimental time. For subject 2, the AM-ULA was administered in all four testing sessions, but the test in the end of stage 3 session could not be scored because the video data was lost.

#### Magnetic table test

In this test, the subject was blind-folded and wore noise-cancelling headphones and earbuds that played white noise. A magnetic table with 10 randomly arranged magnetic blocks was placed in front of the subject. The subject’s task was to locate and remove as many blocks as possible from the table within 2 minutes^18^. Outcomes are how many blocks were removed from the table (Successes), how many unsuccessful removal attempts were made (Errors), and how many errors were detected and successfully corrected (Corrections).

#### Foam block discrimination test

In this test, the subject was blind-folded and wore noise-cancelling headphones with earbuds that played white noise. The subject was presented with a foam block and asked to identify either its size or its compliance^25,26^. When asked to identify size, all blocks in the set were the same compliance and were either small, medium, or large in size. When asked to identify compliance, all blocks in the set were the same size and were either soft, medium, or hard in compliance. The outcome metric was the number of correctly identified blocks. Note that 33% correct is chance. Data from the size and compliance discrimination tasks were combined for comparisons across sessions because there was no significant difference in performance by task condition for either subject (S1: p = 0.43; S2: p = 0.63).

### User Experience Measures

#### Rubber Hand Illusion (RHI) Embodiment Survey

This survey asks the subject to rate their agreement with nine statements on a 7 point Likert scale^18,50,51^. Three of the statements are related to embodiment of the prosthetic (for example, “I felt as if the prosthetic hand was my hand”; “I felt the touch of the objects on the prosthetic hand”.) and six of the statements are controls (for example, “It felt as if I had three arms”.) The subject filled out this survey after performing the functional measures with sensation enabled.

#### Patient Experience Measure (PEM)

This measure, developed for this study, consists of five subscales. Each scale uses a 5 point-Likert scale for subjects to indicate whether they strongly disagree to strongly agree. Two versions of this survey were administered: the long-form was administered in the testing sessions and the short-form was administered daily. In the long-form, the Self-efficacy subscale asks subjects to rate their confidence using the prosthesis to complete 7 items which are typically challenging for upper limb prosthesis users. The Embodiment subscale consists of 8 items that ask about prosthetic embodiment (e.g. the prosthesis is a part of me). The 9-item Body image subscale asks about impact of the prosthesis on self-image (e.g. when I remove my prosthesis I feel more confident). The 3-item Prosthesis Efficiency scale includes items relating to speed and focus required to use the prosthesis. Finally, the Social Touch subscale consists of 11 items pertaining to prosthesis use in social situations. In the short-form, the Self-efficacy subscale consists of 4 items derived from the long-form Self-efficacy subscale. The Embodiment subscale consists of 3 items derived from the long-form of the Embodiment subscale. The Efficiency subscale consists of 1 item derived from the long-form of the Efficiency subscale. The Social Touch subscale consists of 1 item derived from the long-form of the Social Touch subscale. There was no short form of the Body Image subscale. Based on subject responses in this study, we found that the correlation between average response scores on the long-form and the short-form of the PEM was high for all subscales except social interaction (self-efficacy: r = 0.88, both subjects; embodiment: S1: r = 0.91, S2: r = 0.92; social interaction: S1: r = 0.73, S2: r = 0.42; prosthesis efficiency: S1: r = 0.75, S2: r = 0.95).

See the end of the supplement for the PEM surveys. To use the PEM long-form, compute scale scores by averaging the ratings on the items within each scale. The self-efficacy subscale includes items 1.1–1.7 and 3.22–3.24. The embodiment subscale includes items 3.1–3.3, 3.5, 3.6, 3.9, 3.11 and 3.12. The social touch subscale includes items 2.1–2.11. The body image subscale includes items 3.4, 3.7, 3.8, 3.10, 3.13–3.17, and 3.21. The prosthesis efficiency subscale includes items 3.18–3.20. Responses are converted into a 5-pt Likert scale, where strongly disagree is given a rating of 1 and strongly agree is given a rating of 5. Any “not applicable” responses are not scored. The following items are reversed scored (strongly disagree = 5, strongly agree = 1): 2.11, 3.4, 3.7, 3.10, 3.14, and 3.16–3.21. Scale scores are computed by averaging the ratings on all items while omitting any “not applicable” responses from the calculation.

For the PEM short-form, the self-efficacy sub-scale includes items 1.2, 1.6, 1.7, and 1.8. The embodiment sub-scale includes items 1.3, 1.5, and 1.9. The social touch sub-scale includes item 1.1. The prosthesis efficiency sub-scale includes item 1.4. All items are scored as strongly disagree = 1 and strongly agree = 5, except for item 1.5, which is reverse scored. Scale scores are computed by averaging the ratings on all items while omitting any “not applicable” responses from the calculation.

#### Patient-Specific Functional Scale (PSFS)

The PSFS measures activity limitation and functional outcome for people with an orthopedic condition^52–54^. Participants were asked to identify up to three important activities that they are unable to do or have difficulty doing. Then in each lab session, the participant rated their perceived ability to perform the activity on a scale from 0 to 10 where 0 is “unable to perform activity” and 10 is “able to perform activity at the same level as before injury or problem”. The MDC-90 is 2.34 points for upper limb prosthesis users who reported four items (results unpublished, calculated by members of our investigative team using data from another study)^29^.

#### Quick Disabilities of the Arm, Shoulder, and Hand (QuickDASH)

The Quick-DASH is an 11-item survey designed to assess persons with upper extremity musculoskeletal disorders. The Quick-DASH consists of six questions that ask the participant to rate the difficulty of performing different tasks, one question about interference with social interactions, one question about feeling limited due to their disorder, two questions about severity of pain, and one question about sleep difficulty due to pain^55,56^. Each item is rated on a 5-pt Likert scale and all ratings are combined into a single score reflecting perceived disability, where higher scores reflect higher perceived disability. A study on upper limb amputees found the MDC-90 to be 13.9 points^30^.

#### Functional Task Confidence

Immediately before each trial of the magnetic table test and the foam block discrimination task, the subject was asked to rate his confidence on his ability to perform the task^18,25^. For the magnetic table task, confidence was expressed as the number of blocks that the subject predicted that he would be able to move within the allotted time. For the foam block test, confidence was expressed as the percentage of blocks that the subject predicted he would be able to correctly identify.

#### Orthotics Prosthetics Users Survey (OPUS) Upper Extremity Functional Status (UEFS) Task Difficulty

We utilized a modified 28 item version of the OPUS Upper Extremity Functional Scale (UEFS)^37^, a measure of everyday activity performance for use with upper limb adult amputees. The 28 items included the original items and additional items as tested by Jarl (peel potatoes (or fruit) with a knife/peeler, open a bag of chips, take banknote out of the wallet, twist a lid off a small drink bottle, and sharpen a pencil) were added to the new version^38^. In our modified version the response categories were reduced from 5 categories to 4 by removing “cannot perform activity,” and respondents only completed difficulty ratings of items if they had performed the task with their prosthesis. For these reasons, neither the original nor the Resnik modified UEFS scoring algorithms^54^ were appropriate for scoring the iSens version.

We used previously collected calibration data (that used a 22 item UEFS) from a group of 87 prosthesis users with unilateral upper limb amputation from 2 prior studies to simulate the formatting of the iSens modified UEFS. WINSTEPS was utilized to perform IRT analysis on the simulation data set. After examining rating scale category structure, person and item statistics and misfit order and removing poorly fit persons and items, 56.5% of the variance was explained by the measure. Given these findings, we used the revised simulated calibration data to generate a scoring algorithm for the modified UEFS by mapping item difficulty as determined through Rasch analysis on a keyform scaled from 0–100. This key form was used to score the modified UEFS.

#### OPUS Health Quality of Life Index

The OPUS quality of life survey consists of 23 items related to perceived limitations due to the prosthetic, restrictions in abilities, the attitudes or reactions of others, activity interference due to emotional problems, and various aspects of the participant’s emotional state^37^. Each item is rated on a 5-pt Likert scale and all items are combined into a single quality of life score. A study on a group of 67 prosthetics and orthotics wearers, 47 of whom were upper limb prosthesis users, found the MDC to be 7.4^31^.

#### Phantom experience survey

The phantom experience survey consisted of questions about the intensity and modality of their phantom sensation and questions about the position of their phantom. To indicate the perceived length of their phantom limb, participants drew a line on an image of an arm to indicate the most distal point that they perceived their phantom fingertips. The distance between the most distal point on the image, which corresponds to the length of their contralateral limb (or the true length of their amputated limb, if it were present), and the line drawn by the participant was measured using a ruler. Anthropometric data on average male arm length were utilized to convert the measurements into anatomically-relevant lengths^57^.

#### Sensation experience survey

For each stimulation channel, participants drew the location of their percept on an image of a hand each day. All percept locations reported for a given channel were overlaid across days to determine the average percept location. Participants also rated the extent to which 19 words described the modality of the evoked sensory percept on a scale of 0 (did not describe the sensation at all) to 10 (described the sensation very well). This survey was completed daily. Ratings within each day were summed and then the percentage of the percept described by each individual descriptor was calculated. Averages were taken across days, channels, and subjects.

#### Participant interviews

In the End of Stage 2 testing session, participants were interviewed about their experiences with the sensory restoration at home. Interviews were videotaped and transcribed.

### Data analyses

For each metric in the left column of Table 1, One-Way ANOVAs with Tukey pairwise comparisons were run across stages. The diary reports of wear time were pooled across stages and compared to 4 hrs using one-sample t-tests. The reports of wear time were compared between the daily diary reports and the stimulator logs using paired t-tests. For each metric in the right column of Table 1, results were pooled across testing sessions and a two-sample t test was used to compare performance with and without sensation. The data was also pooled within individual sessions, and two-sample t tests were used to compare performance with and without sensation within individual sessions. For the functional tests in the middle column of Table 1, percentage change metrics were calculated by subtracting performance without sensation (OFF) from performance with sensation (ON) and dividing by the maximum possible score on the metric (test max, where test max = 10 for the magnetic table test, 100% for the foam block test, and 40 for the AM-ULA; Because the nine hole peg and clothespin relocation tests are not bounded, test max was taken to be the maximum reported value across all sessions and subjects). The percentage change metric was calculated as ((ON-OFF)/(test max))*100 (Note: Because faster completion times on the nine hole peg test (lower values) indicate better performance, the percentage change due to sensation for this test was calculated as ((OFF-ON)/(test max))*100). For the user experience metrics in the middle column of Table 1, the statistical tests used depended on the measure. The PSFS, QuickDASH, and OPUS quality of life scores were compared pairwise between successive testing sessions using reported MDC values. For the patient experience survey long-form, paired t tests were used to compare individual item ratings between successive pairs of testing sessions. For the magnetic table test and foam block confidence ratings, One-Way ANOVAs with Tukey pairwise comparisons were run across testing sessions. For the RHI embodiment survey, two-sample t-tests were used to compare ratings on embodiment questions to ratings on control questions within each testing session. All tests were two-tailed and run using Minitab Statistical Software (Minitab, Inc., State College, PA). The alpha level for all statistical tests was set to 0.05.

### Data availability

All data generated and analyzed during the current study is available through data transfer agreement upon reasonable request to D.J.T.

## Electronic supplementary material

Supplementary Information
