# Supplementary material for: Home Use of a Neural-connected Sensory Prosthesis Provides the Functional and Psychosocial Experience of Having a Hand Again
Source: Sci Rep. 2018 Jun 29;8:9866. doi: 10.1038/s41598-018-26952-x (PMC6026118; doi:10.1038/s41598-018-26952-x)
Supplement: Supplementary file 1 — Supplementary Information [file 41598_2018_26952_MOESM1_ESM.pdf]

Home Use of a Neural-connected Sensory Prosthesis Provides the Functional and Psychosocial Experience of Having a Hand Again

Emily L. Graczyk[1,4], Linda Resnik[2,3], Matthew A. Schiefer[1,4], Melissa S. Schmitt[4], Dustin J. Tyler[1,4*]

[1]Department of Biomedical Engineering, Case Western Reserve University, Cleveland, OH, USA.

[2]Providence Veterans Affairs Medical Center, Providence, RI, USA.

[3]Health Services, Policy and Practice, Brown University, Providence, RI, USA.

[4]Louis Stokes Cleveland Veterans Affairs Medical Center, Cleveland, OH, USA.

*Correspondence to:

Dustin J. Tyler, PhD

Department of Biomedical Engineering

Case Western Reserve University

10900 Euclid Ave

Cleveland, Ohio 44106

Email: dustin.tyler@case.edu

Supplementary Information:

Figures S1-S3

Table S1

Patient Experience Measure (long-form)

Patient Experience Measure (short-form)

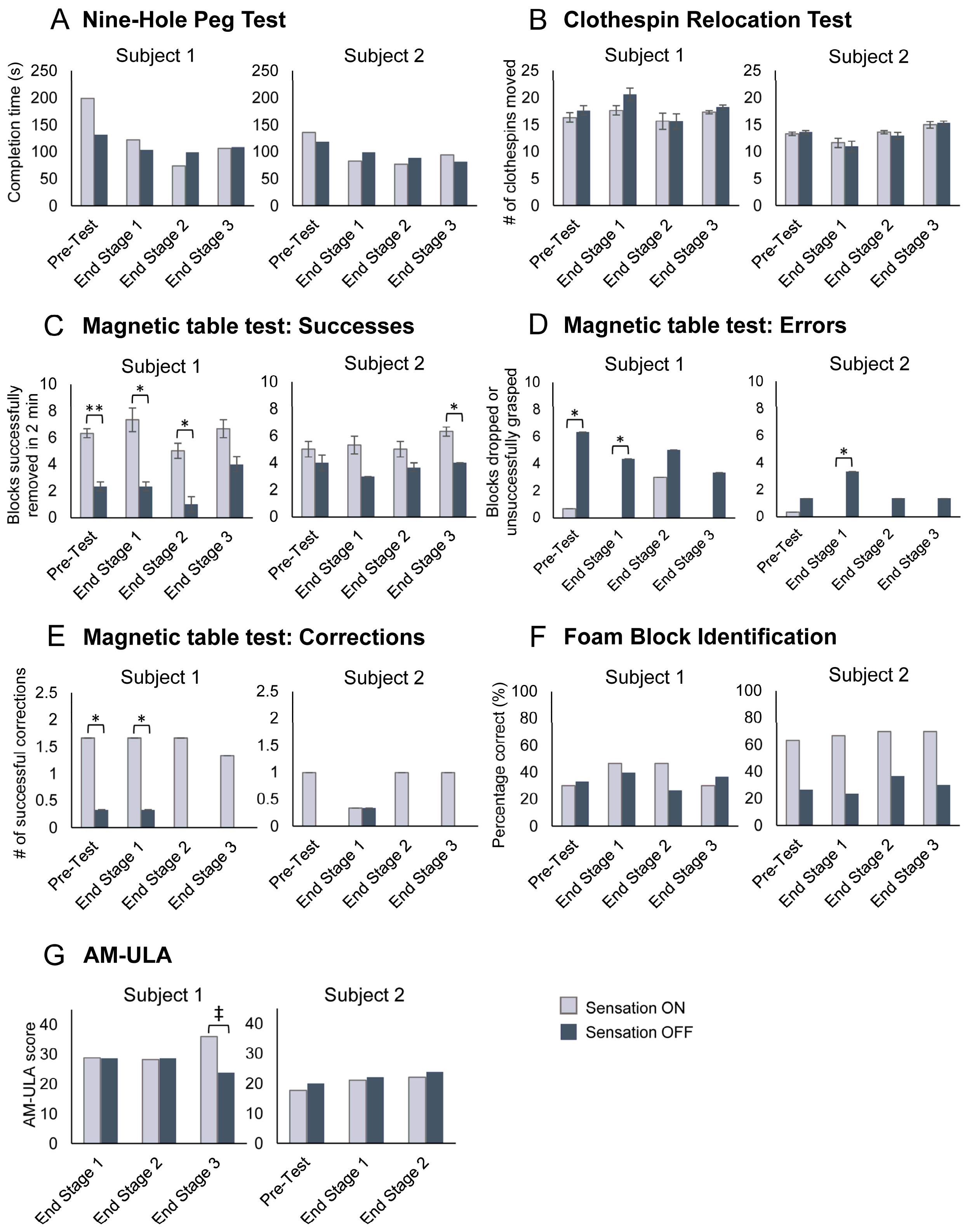

A Nine-Hole Peg Test
Subject 1
Subject 2
Completion time (s)
Pre-Test
End Stage 1
End Stage 2
End Stage 3
B Clothespin Relocation Test
# of clothespins moved
C Magnetic table test: Successes
Blocks successfully removed in 2 min
D Magnetic table test: Errors
Blocks dropped or unsuccessfully grasped
E Magnetic table test: Corrections
# of successful corrections
F Foam Block Identification
Percentage correct (%)
G AM-ULA
AM-ULA score
Sensation ON
Sensation OFF

Figure S1. Raw data from functional performance metrics. A) Nine hole peg test performance by stage and sensation condition for each subject. Faster completion (lower completion time) indicates better performance. B) Clothespin relocation test performance by stage and sensation condition for each subject. More clothespins moved indicates better performance. C) Magnetic table test successes metric by stage and sensation condition for each subject. Greater numbers of successes indicate better performance. D) Magnetic table test errors metric by stage and sensation condition for each subject. Fewer numbers of grasping errors indicate better performance. E) Magnetic table test corrections metric by stage and sensation condition for each subject. Greater numbers of corrections indicate better performance. F) Foam block identification performance by stage and sensation condition for each subject. Higher percentage correct indicates better performance. G) AM-ULA score by stage and sensation condition for each subject. Higher scores indicate better performance. Note: The pre-test session for subject 1 is missing because the test was not performed due to time limitations in that session. The end of stage 3 session for subject 2 is missing because the video data of that session was lost. All panels: Error bars indicate the standard error of the mean. Single asterisk indicates significance of $p<0.05$. Double asterisks indicate significance of $p\leq0.001$. Double cross indicates change greater than the minimal detectable change (MDC-90) reported for that test.

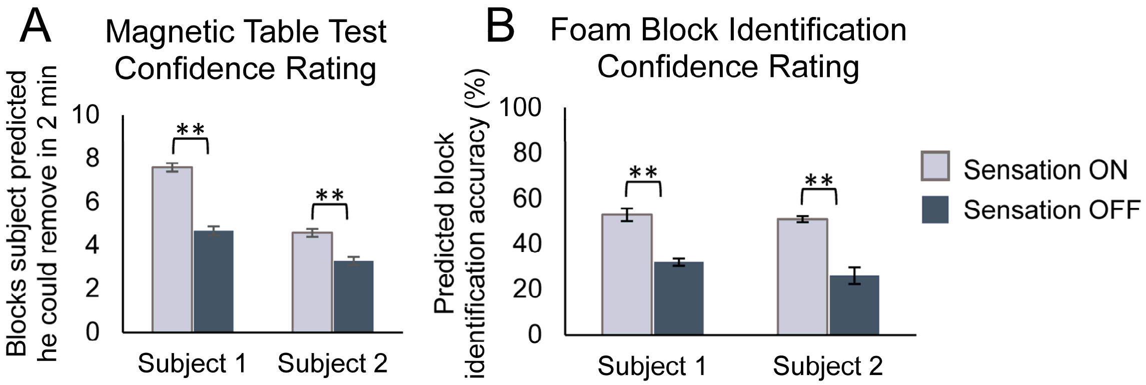

Figure S2. Participant confidence on functional tests performed in the laboratory. A) Confidence on the magnetic table test, measured as the number of blocks that the participant predicted he would be able to successfully remove within the allotted time on the upcoming trial. B) Confidence on the foam block identification test, as measured by the participant's prediction of percentage success on the upcoming trial. All panels: Data was pooled across testing sessions and compared by sensation condition (sensation on vs off) using two-sample t-tests. Error bars indicate standard error of the mean. Double asterisks indicate significance at $p<0.001$.

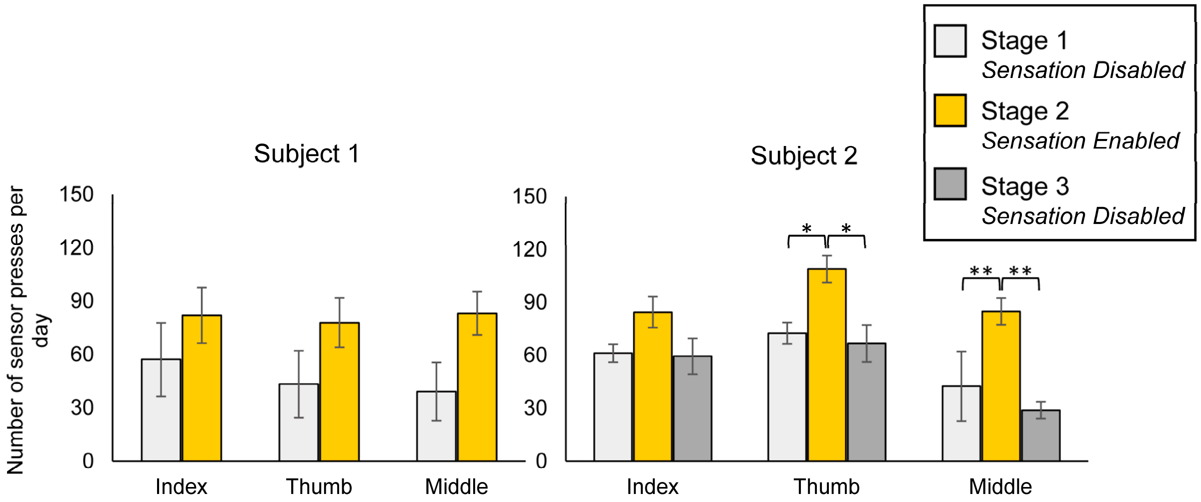

Figure S3. Active usage of the prosthesis for touch or grasp-related activities that involved triggering the pressure sensors. Raw sensor data was analyzed for peaks in force, which indicated instances that the prosthesis was used to touch, manipulate, or grasp objects. Each fingertip sensor (index, thumb, middle finger) was analyzed separately. One-way ANOVAs with Tukey pairwise comparisons were used to compare across study stages. Error bars indicate standard error of the mean. Single asterisk indicates significance of $p<0.05$. Double asterisks indicate significance of $p\leq0.001$. Note that data from stage 3 for subject 1 was not available for analysis due to a malfunction of the stimulator log in stage 2.

Table S1. Modalities of sensory percepts evoked by neural stimulation. Each day, participants reported the extent to which 18 modality words described their sensation. Each descriptor rating was converted into a percentage by dividing by the sum of all descriptor ratings within each stimulation channel each day. The descriptor percentages were averaged across all days, stimulation channels, and participants.

Descriptor	Percentage of Percepts Experienced
touch	24.3%
pressure	20.9%
vibration	16.5%
tingling	15.5%
movement	9.3%
electrical	8.9%
unpleasant	2.8%
edged	1.2%
sharp	0.4%
rough	0.0%
hot	0.0%
cold	0.0%
itchy	0.0%
numb	0.0%
dull	0.0%
cramping	0.0%
throbbing	0.0%
aching	0.0%

CASE WESTERN RESERVE UNIVERSITY<br>CASE SCHOOL OF ENGINEERING	**Patient Experience Measure**
	**Long form**
	**Page 1/2**

Subject #: ____________ Date: ___________ Sensation (circle): **Active/ Inactive**

When you answer the following questions, please consider your experience over the **LAST WEEK**. If you did not perform the activity, make your best guess as to how you would have felt about performing the activity with your prosthesis and with sensation active or inactive, as indicated above.

	1. To what extent do you agree or disagree with the following statements? I was **confident** that I could <u>use my prosthesis to</u>:	Strongly disagree	Disagree	Neither	Agree	Strongly Agree	Not applicable
1.1	Carry a small object (such as a coin) without dropping it.						
1.2	Hold someone else's hand while walking without hurting them						
1.3	Pick up an open plastic water bottle without dropping or crushing it.						
1.4	Drink from a paper cup without dropping or crushing it.						
1.5	Pick up a Ritz cracker without breaking it.						
1.6	Carry a slippery object (such as a silk scarf or tie) without dropping it.						
1.7	Shake hands without hurting or pinching someone.						

	2. To what extent do you agree or disagree with the following statements about <u>using your prosthesis</u> <u>**in the past week**</u>?	Strongly disagree	Disagree	Neither	Agree	Strongly Agree	Not applicable
2.1	I am comfortable using my prosthesis to hold hands with someone close to me.						
2.2	I am comfortable using my prosthesis to shake hands with someone I just met.						
2.3	I am comfortable using my prosthesis to shake hands with someone I know well.						
2.4	I can use my prosthesis to gently squeeze someone else's hand						
2.5	I can feel it when someone else gently squeezes my prosthetic hand.						
2.6	I would use my prosthesis when embracing someone I cared about.						
2.7	I can convey a friendly or caring touch using my prosthesis.						
2.8	I can communicate my anger through touch using my prosthesis.						
2.9	I can use my prosthesis to gently pat a dog or cat.						
2.10	I can control whether I deliver a soft or a firm touch when patting someone on the back using my prosthesis.						
2.11	Wearing my prosthesis interferes with my physical and intimate relationships.						

CASE WESTERN RESERVE UNIVERSITY<br>CASE SCHOOL OF ENGINEERING	**Patient Experience Measure**
	**Long form**
	**Page 2/2**

Subject #: ___________ Date: ____________ Sensation (circle): **Active/ Inactive**

	3. To what extent do you agree or disagree with the following statements about <u>using your prosthesis</u> **<u>in the past week</u>**?	Strongly disagree	Disagree	Neither	Agree	Strongly Agree	Not applicable
3.1	My prosthesis is a part of me.						
3.2	I feel more complete when wearing my prosthesis.						
3.3	My prosthesis felt like it was my hand.						
3.4	I look forward to removing my prosthesis so I can be more comfortable.						
3.5	My prosthesis is an extension of me.						
3.6	I use my prosthesis to express myself.						
	When I remove my prosthesis, I feel:						
3.7	- More confident about myself						
3.8	- Less confident about myself						
3.9	- Sense of loss						
3.10	- Happy						
3.11	- Less whole						
3.12	- More whole						
3.13	- Different from others						
3.14	- Relieved						
3.15	- More shy in public						
3.16	- Less shy in public						
3.17	- More free						
3.18	Using my prosthesis required a lot of focus.						
3.19	Using my prosthesis is much slower than using my other hand.						
3.20	I avoided using my prosthesis to do things because it slowed me down.						
3.21	I looked forward to removing my prosthesis.						
3.22	I felt confident using my prosthesis to pick up fragile objects.						
3.23	I felt confident using my prosthesis to do two-handed tasks.						
3.24	I was willing to try new tasks with my prosthesis.						

CASE WESTERN RESERVE UNIVERSITY CASE SCHOOL OF ENGINEERING	**Patient Experience Measure**
	**Short form**
	**Page 1/1**

Subject #: ___________ Date: ____________ Sensation (circle): **Active/ Inactive**

	1. To what extent do you **agree** or **disagree** with the following statements?	Strongly disagree	Disagree	Neither	Agree	Strongly Agree	Not applicable
1.1	I used my prosthesis to convey a friendly or caring touch.						
1.2	I felt confident using my prosthesis to shake hands.						
1.3	I used my prosthesis to express myself.						
1.4	Using my prosthesis required a lot of focus.						
1.5	I looked forward to removing my prosthesis.						
1.6	I felt confident using my prosthesis to pick up fragile objects.						
1.7	I felt confident using my prosthesis to do two-handed tasks.						
1.8	I was willing to try new tasks with my prosthesis.						
1.9	My prosthesis felt like it was my hand.						
